# Supplementary material for: Discovery and Validation of Lmj_04_BRCT Domain, a Novel Therapeutic Target: Identification of Candidate Drugs for Leishmaniasis
Source: Int J Mol Sci. 2021 Sep 28;22(19):10493. doi: 10.3390/ijms221910493 (PMC8508789; doi:10.3390/ijms221910493)
Supplement: Supplementary file 1 [file ijms-22-10493-s001.zip › ijms-1334907-supplementary.pdf]

Discovery and Validation of Lmj_04_BRCT Domain, a Novel Therapeutic Target: Identification of Candidate Drugs for Leishmaniasis

José Peña-Guerrero [1], Celia Fernández-Rubio [1], Aroia Burguete-Mikeo [1], Rima El-Dirany [1], Alfonso T. García-Sosa [2,*] and Paul Nguewa [1,*]

[1] Department of Microbiology and Parasitology, ISTUN Instituto de Salud Tropical, IdiSNA,
Instituto de Investigación Sanitaria de Navarra, Universidad de Navarra, E-31008 Pamplona, Spain;
jpena.1@alumni.unav.es (J.P.-G.); cfdezrubio@unav.es (C.F.-R.); aburguetem@unav.es (A.B.-M.);
reldirany@alumni.unav.es (R.E.-D.)

[2] Department of Molecular Technology, Institute of Chemistry, University of Tartu, 50411 Tartu, Estonia

* Correspondence: alfonsog@ut.ee (A.T.G.-S.); panguewa@unav.es (P.N.);
Tel.: +372-737-5270 (A.T.G.-S.); +34-948-425-600 (ext. 6434) (P.N.)

Supplementary Materials

Table S1. Summary of currently available therapeutic options against leishmaniasis

Drug	Mechanism of action	Indications	Disadvantages	References
Pentavalent antimonials	Interaction with sulfhydryl-containing molecules	Intralesional (CL) Systemic (MCL)	Local irritation, cardiac and hepatic alterations, anorexia, nausea, and vomiting	[1]
Amphotericin B	Parasite membrane disruption	Parenteral (VL, and HIV co-infected, pregnant, and transplanted patients)	Fever, hypokalemia, myocarditis, and nephrotoxicity	[2][3][4,5]
Miltefosine	Alteration of signaling pathways Apoptosis-like cell death Immunomodulatory properties	Oral (Declining due to emerging resistances, combinatorial therapies)	Gastrointestinal alterations, hepatotoxicity and teratogenic effects	[6][7–11] [12][13][14]
Paromomycin	Hinders protein synthesis and respiration	Intramuscular (VL, India)	Discomfort on application site, low hepato- and ototoxicity	[1][5][15]
Pentamidine	Inhibition parasite DNA synthesis	Intramuscular (complementary for HIV co-infected patients)	Pancreatitis, diabetes mellitus, hypoglycemia, hypotension, cardiac alterations, and hyperkalemia	[16,17][14][1]
Azoles	Inhibition of parasite sterol 14α-demethylase (CYP51)	Combination therapies	Hepato- and cardiotoxicity	[18][19][20,21][22]

**Table S2. Previous approaches on the inhibition of tandem BRCT domains.**		
Target	Description	References
BACH1 binding site of BRCT(BRCA1)	Libraries were explored using high throughput fluorescence assays. The pSXXF motif was annotated as the structural minima for BRCT PPI inhibitor design.	[23][24,25]
BRCT(BRCA1), BRCT(MDC1) and BRCT(TopBP1)	Nanomolar affinity with BRCT(BRCA1) using peptidomimetic inhibition strategy was achieved. A conserved hydrophobic cluster (VLPF) relevant for inhibitor design was discovered.	[26][27]
BRCT(BRCA1)	A peptidomimetic BRCT(BRCA1) inhibition strategy was used to generate an effect similar to BRCA1-knockdown in vitro. This discovery opened new therapeutic opportunities as BRCA1-knockdown cells are sensitive to PARP inhibitor olaparib. However, used inhibitors were labile and had short half-lives. Consequently, there was a need for improvement the stability of BRCT binders.	[28]
BRCT(BRCA1)	Discovery of the first cell permeable BRCT(BRCA1) protein interaction inhibitor, which also displayed synergism with Olaparib, etoposide, and sensitized treated cells to apoptosis induced by ionizing radiation	[29]
BRCT(BRCA1)	Discovery of drug-like BRCT(BRCA1) inhibitor bractoppin, which interacted with BRCT in a similar way than the consensus phosphopeptide. Bractoppin was also highly selective for BRCT (BRCA1) and caused similar effects in the treated cell than the previously cited peptide inhibitors.	[30]

Table S3. Primers used in this work.		
**Target**	**Type**	**Sequence**
GAPDH	Fw	CATCAAGTGCGTGAAGGCGC
	Rv	CGTCGGCGAGTACTCGTGCTG
ABC Transporter A3	Fw	ACGGGAACGGTAACATTGCT
	Rv	GGCACAGCATCGAAATCGTC
ABC transporter C2	Fw	GCAGCCCCATGATGTTTATT
	Rv	TCCGTTGCCTTCACTAGCTT
ABC transporter C6	Fw	TGTCCTCTCAACACGCATCC
	Rv	TCGCAGAGCTCTTCAGTTGG
ABC transporter G4	Fw	TTGGTATCCCCGGCATTCTG
	Rv	AGCAGCACCACAAAGGGATT
ABC transporter G6	Fw	AGCGCAAGGTGAAAAGCAAG
	Rv	CTCCTCGACGGTCACATAGC
ABC transporter H1	Fw	CGGGTTTGTCTTTCAGTCGT
	Rv	CACCAGAGAGCATTGATGGA
APG9	Fw	TCACTCTCGTTTGGTGGCTC
	Rv	AAAGGTCGTCGTGATGTGCT
Cyclin (CYCA)	Fw	CCCCAACACCGCTGACTAAT
	Rv	TCCGACTGGCGGTCTATGTA
Cyclin 6	Fw	AGTACCCTGCACGCCTACTA
	Rv	TTGTTGTTGGCGCAGGAAAG
His-Lys-N	Fw	ACGCTAGAGTGCCGAAGAAG
	Rv	ACACTTCGCACCCGTCATAA
Hsp70	Fw	CAAGGGTAAGAACCTGGCGT
	Rv	GATGGTGGCCTGGAAGTCAA
MCM4	Fw	CGAGTTCGACAAGATGAACG
	Rv	ATTCCACTGTGAGTCCTTCG
MRPA	Fw	ATGGCGACACCAGACTTTGT
	Rv	CTGCGAGGGAGCATGGTTTA
PCNA	Fw	AGATGGACTACCGCAGCA
	Rv	CTCTGATTTCACCTCCGACTTG
PPG3	Fw	CTGGAGAACGTATCCTTTGC
	Rv	ACCTACCGTCTGTCCACG
PRP1	Fw	CTCATGCGTCAGTGCAAGTG
	Rv	AAACAACGGGCAAAAAGCGA
TOP2	Fw	AGTATAAGAAGCTCACCCCG
	Rv	GTTGTTGATGTTGTCTGCCG
Yip1	Fw	AAGCTCCTTGGCAGCAAGAT
	Rv	TGTGTCGGAAAAAGCGCAAG
α-Tubulin	Fw	ATGCGTGAGGCTATCTGCATCCACAT
	Rv	TAGTGGCCACGAGCGTAGTTGTTCG

References

1. Roatt, B.M.; de Oliveira Cardoso, J.M.; De Brito, R.C.F.; Coura-Vital, W.; de Oliveira Aguiar-Soares, R.D.; Reis, A.B. Recent Advances and New Strategies on Leishmaniasis Treatment. *Appl. Microbiol. Biotechnol.* 2020, 1–13.
2. Sundar, S.; Chakravarty, J. Leishmaniasis: An Update of Current Pharmacotherapy. *Expert Opin. Pharmacother.* 2013, *14*, 53–63.
3. Gray, K.C.; Palacios, D.S.; Dailey, I.; Endo, M.M.; Uno, B.E.; Wilcock, B.C.; Burke, M.D. Amphotericin Primarily Kills Yeast by Simply Binding Ergosterol. *Proc. Natl. Acad. Sci. U. S. A.* **2012**, *109*, 2234–2239, doi:10.1073/pnas.1117280109.
4. Sundar, S.; Chakravarty, J.; Rai, V.K.; Agrawal, N.; Singh, S.P.; Chauhan, V.; Murray, H.W. Amphotericin B Treatment for Indian Visceral Leishmaniasis: Response to 15 Daily versus Alternate-Day Infusions. *Clin. Infect. Dis.* **2007**, *45*, 556–561, doi:10.1086/520665.
5. Sundar, S.; Jha, T.K.; Thakur, C.P.; Sinha, P.K.; Bhattacharya, S.K. Injectable Paromomycin for Visceral Leishmaniasis in India. *N. Engl. J. Med.* **2007**, *356*, 2571–2581, doi:10.1056/NEJMoa066536.
6. Croft, S.L.; Neal, R.A.; Pendergast, W.; Chan, J.H. The Activity of Alkyl Phosphorylcholines and Related Derivatives against Leishmania Donovani. *Biochem. Pharmacol.* **1987**, *36*, 2633–2636, doi:10.1016/0006-2952(87)90543-0.
7. Hilgard, P.; Klenner, T.; Stekar, J.; Nössner, G.; Kutscher, B.; Engel, J. D-21266, a New Heterocyclic Alkylphospholipid with Antitumour Activity. *Eur. J. Cancer Part A* **1997**, *33*, 442–446, doi:10.1016/S0959-8049(97)89020-X.
8. Zeisig, R.; Rudolf, M.; Eue, I.; Arndt, D. Influence of Hexadecylphosphocholine on the Release of Tumor Necrosis Factor and Nitroxide from Peritoneal Macrophages in Vitro. *J. Cancer Res. Clin. Oncol.* **1995**, *121*, 69–75, doi:10.1007/BF01202215.
9. Wadhone, P.; Maiti, M.; Agarwal, R.; Kamat, V.; Martin, S.; Saha, B. Miltefosine Promotes IFN-γ-Dominated Anti-Leishmanial Immune Response. *J. Immunol.* **2009**, *182*, 7146–7154, doi:10.4049/jimmunol.0803859.
10. Hochhuth, C.H.; Vehmeyer, K.; Eibl, H.; Unger, C. Hexadecylphosphocholine Induces Interferon-γ Secretion and Expression of GM-CSF MRNA in Human Mononuclear Cells. *Cell. Immunol.* **1992**, *141*, 161–168, doi:10.1016/0008-8749(92)90135-C.
11. Eue, I. Hexadecylphosphocholine Selectively Upregulates Expression of Intracellular Adhesion Molecule-1 and Class I Major Histocompatibility Complex Antigen in Human Monocytes. *J. Exp. Ther. Oncol.* **2002**, *2*, 333–336, doi:10.1046/j.1359-4117.2002.01048.x.
12. Verma, N.K.; Dey, C.S. Possible Mechanism of Miltefosine-Mediated Death of Leishmania Donovani. *Antimicrob. Agents Chemother.* **2004**, *48*, 3010–3015, doi:10.1128/AAC.48.8.3010-3015.2004.
13. Srivastava, S.; Mishra, J.; Gupta, A.K.; Singh, A.; Shankar, P.; Singh, S. Laboratory Confirmed Miltefosine Resistant Cases of Visceral Leishmaniasis from India. *Parasites and Vectors* **2017**, *10*, 1–11, doi:10.1186/s13071-017-1969-z.
14. Sundar, S.; Chakravarty, J. An Update on Pharmacotherapy for Leishmaniasis. *Expert Opin. Pharmacother.* 2015, *16*, 237–252.
15. Kim, D.H.; Chung, H.J.; Bleys, J.; Ghohestani, R.F. Is Paromomycin an Effective and Safe Treatment against Cutaneous Leishmaniasis? A Meta-Analysis of 14 Randomized Controlled Trials. *PLoS Negl. Trop. Dis.* **2009**, *3*, e381, doi:10.1371/journal.pntd.0000381.
16. Nguewa, P.A.; Fuertes, M.A.; Cepeda, V.; Iborra, S.; Carrión, J.; Valladares, B.; Alonso, C.; Pérez, J.M. Pentamidine Is an Antiparasitic and Apoptotic Drug That Selectively Modifies Ubiquitin. *Chem. Biodivers.* **2005**, *2*, 1387–1400, doi:10.1002/cbdv.200590111.
17. Yang, G.; Choi, G.; No, J.H. Antileishmanial Mechanism of Diamidines Involves Targeting Kinetoplasts. *Antimicrob. Agents Chemother.* **2016**, *60*, 6828–6836, doi:10.1128/AAC.01129-16.

18. Saccoliti, F.; Madia, V.N.; Tudino, V.; De Leo, A.; Pescatori, L.; Messore, A.; De Vita, D.; Scipione, L.; Brun, R.; Kaiser, M.; et al. Biological Evaluation and Structure-Activity Relationships of Imidazole-Based Compounds as Antiprotozoal Agents. *Eur. J. Med. Chem.* **2018**, *156*, 53–60, doi:10.1016/j.ejmech.2018.06.063.
19. Pandharkar, T.; Zhu, X.; Mathur, R.; Jiang, J.; Schmittgen, T.D.; Shaha, C.; Werbovetz, K.A. Studies on the Antileishmanial Mechanism of Action of the Arylimidamide DB766: Azole Interactions and Role of CYP5122A1. *Antimicrob. Agents Chemother.* **2014**, *58*, 4682–4689, doi:10.1128/AAC.02405-14.
20. Emad, M.; Hayati, F.; Fallahzadeh, M.K.; Namazi, M.R. Superior Efficacy of Oral Fluconazole 400 Mg Daily versus Oral Fluconazole 200 Mg Daily in the Treatment of Cutaneous Leishmania Major Infection: A Randomized Clinical Trial. *J. Am. Acad. Dermatol.* **2011**, *64*, 606–608, doi:10.1016/j.jaad.2010.04.014.
21. Alrajhi, A.A.; Ibrahim, E.A.; De Vol, E.B.; Khairat, M.; Faris, R.M.; Maguire, J.H. Fluconazole for the Treatment of Cutaneous Leishmaniasis Caused by *Leishmania Major*. *N. Engl. J. Med.* **2002**, *346*, 891–895, doi:10.1056/NEJMoa011882.
22. Galvão, E.L.; Rabello, A.; Cota, G.F. Efficacy of Azole Therapy for Tegumentary Leishmaniasis: A Systematic Review and Meta-Analysis. *PLoS One* **2017**, *12*, e0186117, doi:10.1371/journal.pone.0186117.
23. Lokesh, G.L.; Muralidhara, B.K.; Negi, S.S.; Natarajan, A. Thermodynamics of Phosphopeptide Tethering to BRCT: The Structural Minima for Inhibitor Design. *J. Am. Chem. Soc.* **2007**, *129*, 10658–10659, doi:10.1021/ja0739178.
24. Lokesh, G.L.; Rachamallu, A.; Kumar, G.D.K.; Natarajan, A. High-Throughput Fluorescence Polarization Assay to Identify Small Molecule Inhibitors of BRCT Domains of Breast Cancer Gene 1. *Anal. Biochem.* **2006**, *352*, 135–141, doi:10.1016/j.ab.2006.01.025.
25. Simeonov, A.; Yasgar, A.; Jadhav, A.; Lokesh, G.L.; Klumpp, C.; Michael, S.; Austin, C.P.; Natarajan, A.; Inglese, J. Dual-Fluorophore Quantitative High-Throughput Screen for Inhibitors of BRCT-Phosphoprotein Interaction. *Anal. Biochem.* **2008**, *375*, 60–70, doi:10.1016/j.ab.2007.11.039.
26. Yuan, Z.; Kumar, E.A.; Kizhake, S.; Natarajan, A. Structure-Activity Relationship Studies to Probe the Phosphoprotein Binding Site on the Carboxy Terminal Domains of the Breast Cancer Susceptibility Gene 1. *J. Med. Chem.* **2011**, *54*, 4264–4268, doi:10.1021/jm1016413.
27. Yuan, Z.; Kumar, E.A.; Campbell, S.J.; Palermo, N.Y.; Kizhake, S.; Glover, J.N.M.; Natarajan, A. Guided Inhibitor Design. **2011**, 764–767.
28. Pessetto, Z.Y.; Yan, Y.; Bessho, T.; Natarajan, A. Inhibition of BRCT(BRCA1)-Phosphoprotein Interaction Enhances the Cytotoxic Effect of Olaparib in Breast Cancer Cells: A Proof of Concept Study for Synthetic Lethal Therapeutic Option. *Breast Cancer Res. Treat.* **2012**, *134*, 511–517, doi:10.1007/s10549-012-2079-4.
29. Na, Z.; Pan, S.; Uttamchandani, M.; Yao, S.Q. Discovery of Cell-Permeable Inhibitors That Target the BRCT Domain of BRCA1 Protein by Using a Small-Molecule Microarray. *Angew. Chemie - Int. Ed.* **2014**, *53*, 8421–8426, doi:10.1002/anie.201405169.
30. Periasamy, J.; Kurdekar, V.; Jasti, S.; Nijaguna, M.B.; Boggaram, S.; Hurakadli, M.A.; Raina, D.; Kurup, L.M.; Chintha, C.; Manjunath, K.; et al. Targeting Phosphopeptide Recognition by the Human BRCA1 Tandem BRCT Domain to Interrupt BRCA1-Dependent Signaling. *Cell Chem. Biol.* **2018**, *25*, 677-690.e12, doi:10.1016/j.chembiol.2018.02.012.
